# Comparative nucleic acid transfection efficacy in primary hepatocytes for gene silencing and functional studies

**DOI:** 10.1186/1756-0500-4-8

**Published:** 2011-01-18

**Authors:** Jae-Seung Park, Sneha Surendran, Lisa M Kamendulis, Núria Morral

**Affiliations:** 1 Department of Medical and Molecular Genetics, Indiana University School of Medicine, 975 West Walnut St, IB130, Indianapolis, Indiana 46202, USA; 2 Department of Pharmacology and Toxicology, Indiana University School of Medicine, 635 Barnhill Dr., MS A-401, Indianapolis, Indiana 46202, USA; 3 Department of Biochemistry and Molecular Biology, Indiana University School of Medicine, 635 Barnhill Dr., MS 4053, Indianapolis, Indiana 46202, USA

## Abstract

**Background:**

Primary hepatocytes are the best resource for *in vitro *studies directed at understanding hepatic processes at the cellular and molecular levels, necessary for novel drug development to treat highly prevalent diseases such as non-alcoholic steatohepatitis, cardiovascular disease and type 2 diabetes. There is a need to identify simple methods to genetically manipulate primary hepatocytes and conduct functional studies with plasmids, small interfering RNA (siRNA) or microRNA (miRNA). New lipofection reagents are available that have the potential to yield higher levels of transfection with reduced toxicity.

**Findings:**

We have tested several liposome-based transfection reagents used in molecular biology research. We show that transfection efficiency with one of the most recently developed formulations, Metafectene Pro, is high with plasmid DNA (>45% cells) as well as double stranded RNA (>90% with siRNA or microRNA). In addition, negligible cytotoxicity was present with all of these nucleic acids, even if cells were incubated with the DNA:lipid complex for 16 hours. To provide the proof of concept that these conditions can be used not only for overexpression of a gene of interest, but also in RNA interference applications, we targeted two liver expressed genes, Sterol Regulatory Element-Binding Protein-1 and Fatty Acid Binding Protein 5 using plasmid-mediated short hairpin RNA expression. In addition, similar transfection conditions were used to optimally deliver siRNA and microRNA.

**Conclusions:**

We have identified a lipid-based reagent for primary hepatocyte transfection of nucleic acids currently used in molecular biology laboratories. The conditions described here can be used to expedite a large variety of research applications, from gene function studies to microRNA target identification.

## Background

The liver is a critical tissue that controls carbohydrate and lipid homeostasis. Highly prevalent human conditions, including obesity, cardiovascular disease, the metabolic syndrome, non-alcoholic steatohepatitis (NASH), or type 2 diabetes, are characterized by alterations in hepatic glucose and/or lipid metabolism. Deciphering the molecular mechanisms that mediate metabolic responses will open the possibility for the development of more effective therapies.

Establishing the function of genes and/or validating their role in disease is commonly attained through generation of null alleles. The discovery of RNA interference (RNAi) has provided novel avenues to accelerate gene function studies as well as drug target discovery [1-5]. RNAi is triggered by double stranded RNA (dsRNA) of variable lengths, typically 21 to 28-nucleotides (nt). These can be chemically synthesized molecules (known as small interfering RNA, or siRNA) that upon delivery into cells or tissues, block gene expression for a few days. Re-administration is necessary for a sustained silencing effect. Alternatively, short hairpin RNA (shRNA) can be used to provide a continuous source of silencing molecules. To generate shRNA, expression cassettes are engineered using RNA polymerase II or RNA polymerase III promoters. The shRNA is transcribed in the nucleus as a linear molecule that folds to generate a stem of approximately 21-nt, connected with a loop sequence of variable length. The shRNA hairpin structure is cleaved into a siRNA [6-8]. Once an effective silencing shRNA construct is identified, the expression cassette can be incorporated into viral vectors for delivery to tissues *in vivo *[9].

In recent years it has become clear that microRNA (miRNA) are crucial modulators of biological functions, affecting processes as important as development and cell cycle stage. It has been estimated that more than 30% of human genes are regulated by miRNA at an average of 200 genes per miRNA [10-13]. MicroRNA exert their action by binding to the 3' UTR of mRNA and inhibiting protein synthesis or inducing mRNA degradation [14,15]. Gene target prediction coupled to experimental confirmation is beginning to yield information on the role of miRNA in normal cellular pathways as well as in disease. MicroRNA target identification is typically attained by transfection using chemically synthesized miRNA mimics (acting as mature miRNA that decrease levels of target mRNA and/or protein).

The primary culture of hepatocytes is regarded as the cellular model with highest similarity to liver physiology, and is the preferred approach to perform functional studies. Primary hepatocytes can be easily obtained by enzymatic digestion with collagenase [16-18]. Compared to other methods, transfection with cationic lipids offers the advantage of being a simple method of gene transfer, shortening gene function studies considerably. In recent years a variety of novel lipofection reagents have been developed with the potential to improve nucleic acid delivery in the absence of toxic effects. Optimization experiments in primary cells are highly time-consuming and expensive to perform. Here, we show transfection conditions with DNA and RNA molecules to knock-down two hepatic genes, Sterol Regulatory Element-Binding Protein-1 (SREBP-1) and Fatty Acid Binding Protein 5 (FABP5), using shRNA-expressing plasmids or siRNA. Similar conditions were successfully used to transfect primary hepatocytes with miRNA.

## Results and discussion

### Plasmid transfection optimization

**Figure 1:**
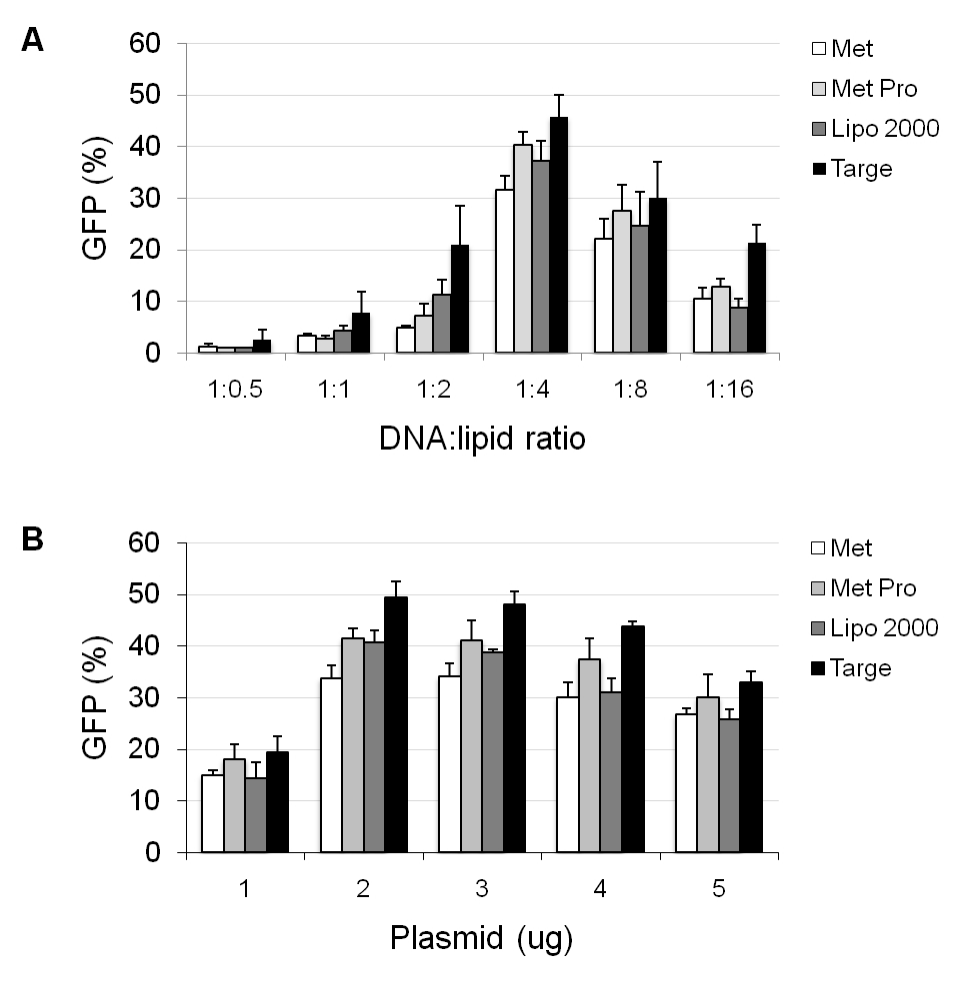
**Effect of DNA:lipid ratio and DNA concentration on transfection efficiency**. Cells were transfected with pRNAT-H1.1/neo GFP plasmid complexed with Metafectene (Met), Metafectene Pro (Met Pro), Lipofectamine 2000 (Lipo 2000) or Targefect (Targe), for 16 hr. (A) DNA:lipid ratio. Two μg DNA was complexed with lipid at ratios of 1:0.5, 1:1, 1:2, 1:4, 1:8, 1:16. The percentage of GFP-positive cells was determined 24 hr after transfection. Values represent the average of 4 independent experiments. (B) DNA concentration. Using a 1:4 DNA:lipid ratio, cells were transfected with 1, 2, 3, 4, and 5 μg of DNA. The percentage of GFP-positive cells was determined 24 hr after transfection. Values represent the average of 3 independent experiments.

Critical aspects of mouse primary hepatocyte isolation by collagenase digestion are the strain and age of the animal, which affect cell viability, even if using the same collagenase concentration and perfusion time (data not shown). Using the protocol described in the Methods section, we consistently obtained high cell yield and viability (85-90%). High transfection efficiency can be obtained in cell lines with essentially all commercial transfection reagents, whereas primary cells in general have proven much more difficult to transfect [19,20]. Plasmid transfection, in particular, is more challenging, as these are large molecules and more difficult to be taken up by cells. To analyze the potential of novel lipofection methods to improve primary hepatocyte gene delivery, a 3 kb plasmid expressing green fluorescent protein (GFP) was complexed to four reagents: Metafectene, Metafectene Pro, Lipofectamine 2000, and Targefect-Hepatocyte. These reagents were selected based on information available from the respective company indicating high transfection efficiency in multiple cell lines and/or primary cells (including primary hepatocytes), in addition to low toxicity. To develop an optimal protocol for transfecting plasmid DNA into primary hepatocytes, DNA:lipid ratio, DNA concentration, culture medium and transfection time were considered. As shown in Figure 1A, we found that transfection was most efficient with all reagents when using a DNA:lipid ratio of 1:4. Targefect yielded the highest transfection efficiency (45.8 ± 4.2%), followed by Metafectene Pro (40.3 ± 2.6%), Lipofectamine 2000 (37.3 ± 3.8%), and Metafectene (31.5 ± 2.9%) (Figure 1A). We then tested the effect of total amount of DNA, by transfecting cells with 1 to 5 μg, and maintaining the 1:4 (DNA:lipid) ratio. The highest transfection efficiency in the absence of toxicity was obtained with 2 μg with all reagents (Figure 1B). Because Metafectene gave the lowest transfection efficiency under all conditions, further optimization experiments were conducted without this reagent.

**Figure 2:**
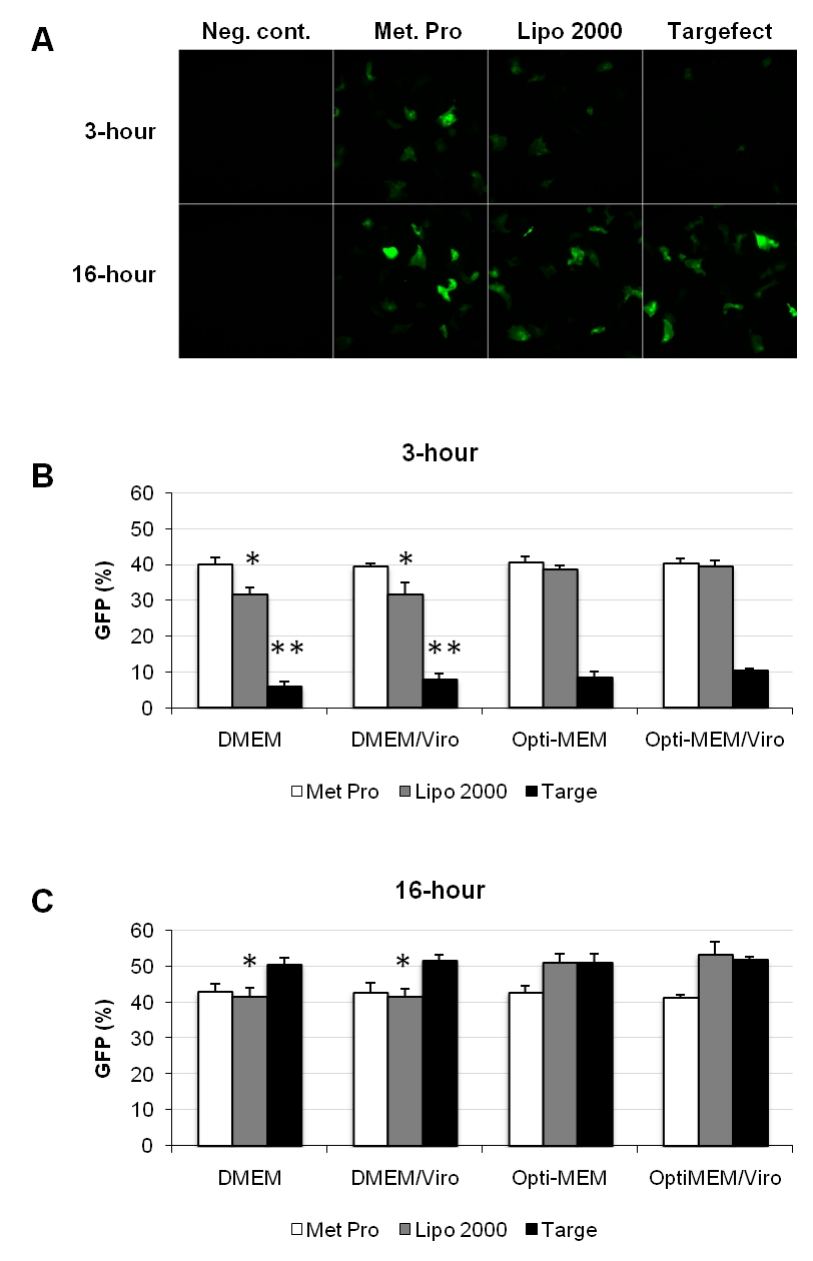
**Influence of culture medium and incubation time on transfection efficiency**. Primary hepatocytes were transfected with 2 μg of plasmid and Metafectene Pro (Met Pro), Lipofectamine 2000 (Lipo 2000) and Targefect (Targe) at a ratio of 1:4. Cells were incubated with DMEM or Opti-MEM for 3 hr or 16 hr. The percentage of GFP-positive cells was determined 24 hr after transfection. (A) GFP images were taken from cells incubated with DMEM media. (B) Quantification of GFP-positive cells after 3 hr transfection in the presence of DMEM or OptiMEM with or without Virofect enhancer. (C) Quantification of GFP-positive cells after 16 hr transfection in the presence of DMEM or OptiMEM with or without Virofect enhancer. Values represent the average of 5 independent experiments. *p < 0.01, **p < 0.05 between DMEM and Opti-MEM, and DMEM/Viro and Opti-MEM/Viro.

To investigate the influence of medium and transfection time, primary hepatocytes were transfected with Metafecte Pro, Lipofectamine 2000 or Targefect in the presence of DMEM (Figure 2A) or Opti-MEM, with/without Virofect enhancer, for 3 hours or 16 hours (Figure 2A, 2B, 2C). The presence of Opti-MEM resulted in significantly higher transfection rates when Lipofectamine 2000 was used (approximately 10% increase at both, 3 and 16 hours) (Figure 2B, 2C), a small but significant increase on cells incubated with Targefect for 3 hours (Figure 2B), and no statistically significant difference with Metafectene Pro (Figure 2B, 2C). Virofect enhancer had no significant effect on transfection efficiency when Metafectene Pro, Lipofectamine 2000 or Targefect were used (Figure 2B, 2C). Interestingly, longer transfection time had a large influence on percentage of GFP-positive cells when Targefect was used. For example, in DMEM-incubated cells, 5.8 ± 1.6% were GFP-positive when transfection lasted 3 hours, while 50.6 ± 1.9% were positive when cells were transfected for 16 hours (p < 0.01). Transfection time also influenced the % of GFP-positive cells when using Lipofectamine 2000. Approximately a 10% increase was observed at 16 hours compared to 3 hours (31.4 ± 2.3% at 3 hours and 41.6 ± 2.5% at 16 hours with DMEM; p < 0.01; 38.6 ± 1.1% at 3 hours and 51.2 ± 2.4% at 16 hours with Opti-MEM; p < 0.01). Transfection time had a negligible effect with Metafectene Pro (39.8 ± 2.2% at 3 hours and 43.0 ± 2.1% at 16 hours, using DMEM; p = 0.046; 40.4 ± 1.8% at 3 hours and 42.8 ± 1.9% at 16 hours using Opti-MEM; p = 0.07).

High efficiency of transfection is often associated with toxicity. Ideally, a transfection reagent should yield high transfection efficiency with minimal or no cytotoxicity. To examine this, primary hepatocytes were cultured in the presence of DMEM or Opti-MEM, using Metafectene Pro, Lipofectamine 2000, and Targefect and cytotoxicity was evaluated after 24, 48, and 72 hr. As shown in Table 1, there was barely any difference in cytotoxicity among transfection reagents at 24 hours post-transfection; however Metafectene Pro resulted in much less cytotoxicity than Lipofectamine 2000 and Targefect at 48 and 72 hours post-transfection. Targefect yielded the highest level of transfection, but was by far the most toxic of the three reagents. Lipofectamine 2000 gave slightly higher levels of transfection than Metafectene Pro and similar levels of toxicity when cells were cultured in the presence of Opti-MEM. However, transfection using Lipofectamine 2000 resulted in lower reproducibility than the other reagents, which was affected by elapsed time between cell plating and transfection (data not shown). Overall, Metafectene Pro gave the most consistent results, with least toxicity and shortest transfection time. This reagent has been previously shown to have superior performance in prostate cancer cells and the human embryonic carcinoma (EC) stem cell line NTERA2 [21]. Our data suggest that Metafectene Pro is a suitable reagent for use in primary hepatocytes, either for short-term expression (< 24 hours) or longer expression (>24 hours).

**Table 1:** Cytotoxic effect induced by transfection reagents

Reagents	Media	Cytotoxicity		
		
		24 hr	48 hr	72 hr
Negative control	DMEM	-	-	-
	Opti-MEM	-	-	-

Metafectene Pro	DMEM	-	+	+
	Opti-MEM	-	+	+

Lipofectamine 2000	DMEM	-	++	+++
	Opti-MEM	-	+	++

Targefect	DMEM	+	+++	++++
	Opti-MEM	-	++	++++

Cytotoxicity was assessed by estimating the percentage of cell density in 6 fields per well, relative to the negative control for each time point. +: < 20%; ++: < 40%; +++: < 60%; ++++: < 80% cell death.

### SREBP-1 and FABP5 silencing in primary hepatocytes

**Figure 3:**
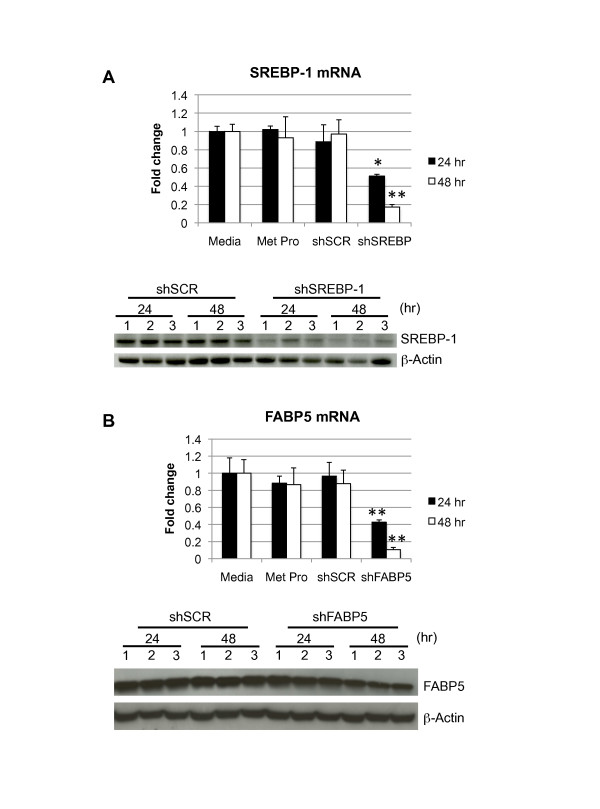
**Gene silencing using plasmid transfection**. Primary hepatocytes cultured in DMEM were transfected with plasmids (2 μg) expressing shRNA to knock-down SREBP1 or FABP5 and Metafectene Pro (1:4 ratio), for 16 hr. (A) SREBP-1 gene silencing; (B) FABP5 gene silencing. The level of mRNA was quantified by real-time RT-PCR (A and B, top). The level of silencing is expressed relative to medium-treated cells. Proteins were quantified by Western blot (A and B, bottom). β-actin was used as a normalizer. Data are representative of two independent experiments (n = 3); *p < 0.05; **p < 0.01 shSCR compared to shSREBP-1 or shFABP5.

To provide the proof of concept that the transfection conditions described above are sufficient to silence an endogenous gene, primary hepatocytes were transfected with plasmids expressing shRNA to knock-down the transcription factor SREBP-1 [9] and the fatty acid binding protein FABP5 [22]. As shown in Figure 3, SREBP-1 mRNA expression was significantly reduced to 58% and 18% (at 24 and 48 hours, respectively) compared to the levels observed in cells treated with the shSCR control plasmid [expressing a scrambled sequence that does not bind to an mRNA, based on Basic Local Alignment Search Tool (BLAST) analysis (National Center for Biotechnology Information, NIH]. Western blot analysis revealed that SREBP-1 protein levels decreased in parallel to mRNA levels. FABP5 mRNA was reduced to 44% and 12% at 24 and 48 hours, respectively, compared to samples treated with shSCR plasmid. FABP5 protein reduction was evident only in samples harvested 48 hours after transfection, suggesting this protein has a long half-life. Thus, when testing the efficacy of custom-designed shRNA sequences, measuring the mRNA of the target gene is a more reliable method to assess the silencing efficacy of the constructs than measuring protein levels. In conclusion, the conditions reported here allow gene silencing of two independent genes by transfection of plasmids expressing shRNA.

**Figure 4:**
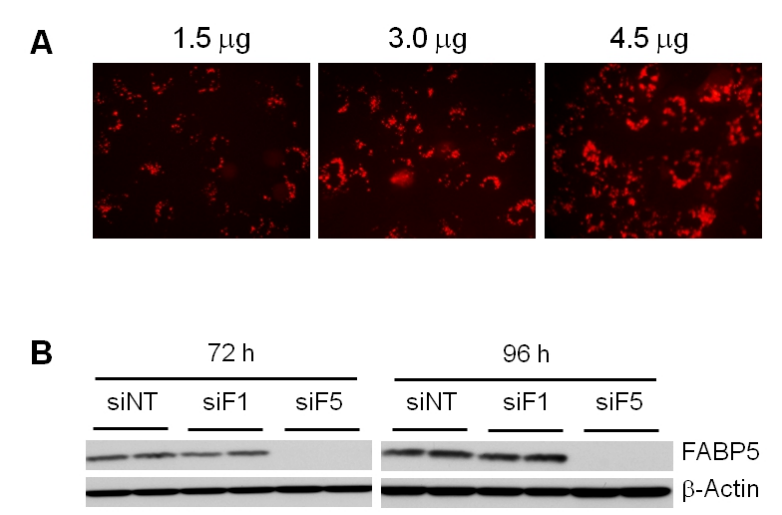
**miRNA and siRNA transfection**. (A) miRNA transfection. Primary hepatocytes were cultured in 6-cm dishes at 1 × 10^6 ^cell/dish and transfected with 1.5, 3.0 and 4.5 μg Dy547-labeled miRIDIAN microRNA Mimic Transfection Control. The percentage of positive cells was determined 24 hr after transfection. (B) siRNA transfection to knock-down FABP5. Cells were cultured in 6-well plates and incubated with 2 μg FABP5 siRNA, FABP1 siRNA or non-targeting siRNA (ratio 1:3). Cells were harvested 72 or 96 hours later. FABP5 levels were analyzed by Western blot. FABP1 is a member of the FABP family. Only the siRNA that is specific for FABP5 reduces its protein levels. siNT, non-targeting siRNA; siF1, FABP1 siRNA; siF5, FABP5 siRNA.

When conducting studies to address the function of a gene, using a chemically synthesized siRNA may be the preferred approach. In these experiments as well as in experiments directed at miRNA target identification, it is important to use conditions that result in highest transduction efficiency, to maximize the level of inhibition on mRNA and/or protein expression. To optimize the transfection conditions using a chemically synthesized double stranded RNA molecule, a fluorescently labeled miRNA, was used. Cells were transfected with 0.5, 1, or 2.5 μg of Dy547-labeled miRIDIAN microRNA Mimic Transfection Control. All conditions resulted in the presence of a fluorescent signal in the cytoplasm in a high percentage of cells (>90%). Using 2.5 μg of miRNA the intensity in the cytoplasm was slightly stronger than in cells transfected with 1 μg, but there were large clumps outside of the cells, suggesting that not all nucleic acid was taken by cells. Experiments were repeated in 6-cm dishes using the same dsRNA:lipid ratio and 1.5, 3.0 and 4.5 μg miRNA. Similar results were observed to those seen in 6-well plates (Figure 4A). No cytotoxicity was observed in any of these conditions for 48 hours.

To test the feasibility of using these conditions for transfection using siRNA, primary hepatocytes were incubated with siRNA to target FABP5, a non-targeting siRNA or with FABP1 siRNA (another member of the FABP family expressed in liver) (Figure 4B). A significant FABP5 knock-down was observed after 72 and 96 hours with the siRNA specific to FABP5. Thus, similar transfection conditions can be used for delivery of large plasmids, miRNA and siRNA in primary hepatocytes.

In addition to be important for gene function studies, plasmid-mediated expression of shRNA in primary hepatocytes has other applications. *In vivo *RNAi offers the possibility to overcome many of the limitations presented by knock-out animal models. For example, absence of a gene may be incompatible with development, leading to death *in utero*. Using viral vector delivery of shRNA-expression cassettes it is possible to induce gene silencing in an adult animal. For the liver, in particular, adenovirus-mediated shRNA expression represents an excellent alternative, as the adenovirus has high tropism for hepatocytes [9]. Using the approach described here, several shRNA-expressing plasmids can be tested for their efficacy at silencing the target gene in primary hepatocytes, prior to transferring the most efficient shRNA expression cassette to an adenoviral vector for *in vivo *studies.

## Conclusion

This study shows efficient transfection of primary hepatocytes and negligible cytotoxicity using plasmid DNA and dsRNA complexed to Metafectene Pro. These conditions can be applied to (i) functional studies where a gene of interest needs to be silenced, (ii) gene overexpression studies, (iii) testing the efficacy of shRNA constructs to target hepatic genes, and (iv) delivery of small RNA molecules, such as siRNA or miRNA mimics, which resulted in >90% transfection efficiency. The primary hepatocyte transfection conditions described here can be used to accelerate gene discovery, drug target validation and miRNA target identification studies, that are urgently needed to treat prevalent human diseases such as obesity, non-alcoholic steatohepatitis, cardiovascular disease, and type 2 diabetes.

## Materials and methods

### Animals

Male C57BL/6J mice (11 to 12 wks old, 26 to 30 g) were purchased from The Jackson Laboratory (Bar Harbor, ME). Mice were fed rodent chow *ad libitum *and allowed free access of water. A standard 12 h light/12 h dark cycle (7 AM/7 PM) was maintained throughout the experiments. Animal studies were performed in compliance with Indiana University School of Medicine Institutional Animal Care and Use Committee guidelines.

### Isolation of mouse primary hepatocytes

Primary hepatocytes were isolated by collagenase digestion, following a previously described protocol [16,17]. Mice were anesthetized by intraperitoneal injection of sodium pentobarbital (90 mg/kg). The portal vein was cannulated with a 22-gauge intravenous catheter. The liver was perfused with Ca^2+^, Mg^2+^-free Hanks' buffer solution (Invitrogen, Carlsbad, CA) containing 0.5 mM ethylene glycol-bis-(2-aminoethylether) *N,N,N',N'*-tetracetic acid (EGTA), and 0.05 M *N*-2-hydroxyethylpiperazine-*N*-2-ethane sulfonic acid (HEPES) (pH 7.4) maintained at 37°C, at a rate of 5 ml/min for 5 min. This was followed by perfusion of the collagenase-containing solution [DMEM (Cellgro, Lawrence, KS) with 0.05% collagenase Type IA (Sigma, St. Louis, MO)] for 5 minutes (5 ml/min), with intermittent obstruction of the inferior vena cava for 15 seconds. The liver was transferred to 10 mm dishes with 15 ml DMEM containing 0.05% collagenase and was mechanically dissociated into single cells. Ten ml DMEM supplemented with 10% heat-inactivated fetal bovine serum (FBS) were added to cells to reduce collagenase activity. Cells were filtered through a 70 μm pore size strainer (BD, San Jose, CA) and centrifuged at 100 × g for 5 min at 10°C. The cell pellets were gently washed with 20 ml DMEM/10% FBS, and centrifuged at 100 × g for 5 min at 10°C. This low centrifugation step allows pelleting hepatocytes while significantly reducing the number of other smaller liver cells [17]. Cells were resuspended in DMEM/10% FBS with antibiotics. Cell viability was assessed by trypan blue dye exclusion (85-90%). Primary hepatocytes were plated at a density of 4 × 10^5 ^cells in 2 ml of DMEM/10% FBS with antibiotics on 6-well plates, which resulted in 90% confluency the day after (Corning, Lowell, MA). Medium was replaced 2 hours after plating.

### Transient transfection

After overnight incubation, plated primary hepatocytes were transfected with plasmid pRNAT-H1.1/neo containing a green fluorescent protein (GFP) expression cassette (GeneScript, Piscataway, NJ). Four transfection reagents were tested: Metafectene (Biontex Laboratories GmbH, Munich, Germany), Metafectene Pro (Biontex Laboratories GmbH), Lipofectamine 2000 (Invitrogen) and Targefect-Hepatocyte (Targeting Systems, El Cajon, CA). Given that each manufacturer recommends different time frames for incubation of cells with DNA/lipid complexes, 3 and 16 hours were used, as these covered the lowest and highest times suggested. DNA and lipid were diluted in 100 μl of medium (in the absence of FBS and antibiotics), in separate tubes. DNA was added to the lipid, gently mixed, and incubated at room temperature for 20 minutes. The complex was added to the cells and the plate was carefully swirled to ensure even distribution. Transfection was conducted in the presence of Dulbecco's modified Eagle's medium (DMEM) or OptiMEM supplemented with 10% FBS, 100 U of penicillin G and 100 μg of streptomycin per ml. In addition, some experiments (as described in the text) included Virofect in the transfection medium. Cells were incubated at 37°C in a 5% CO_2 _incubator. Medium was replaced with DMEM or OptiMEM containing 10% FBS and antibiotics after 3 or 16 hours of transfection. All conditions were tested in duplicate wells and at least in three independent experiments. GFP-positive cells were counted in 6 fields per well on an inverted microscope CKX41 (Olympus, Center Valley, PA) with fluorescence illuminator, BH2-RFL-T3 (Olympus). Fluorescence images were acquired with a Nikon TE 2000 U inverted microscope. Images were acquired with a 40× Air EL WD Plan Fluor 0.60NA DIC objective (Melville, NY), a Hamamatsu 1394 Orca-ER Cooled CCD Camera (Bridgewater, NJ). Images were processed with Image J software (NIH, Bethesda, MA).

Cytotoxicity was assessed by estimating the percentage of cell density in 6 fields per well, relative to the negative control for each time point (+: < 20%; ++: < 40%; +++: < 60%; ++++: < 80% cell death).

The construction of shRNA-expressing plasmids to knock-down SREBP-1 and FABP5 has been previously described [9,22]. For microRNA transfection optimization in 6-well plates, 0.5, 1, or 2.5 μg of Dy547-labeled miRIDIAN microRNA Mimic Transfection Control (Dharmacon, Lafayette, CO) was mixed with Metafectene Pro at a 1:3 ratio, following the same protocol described above for plasmids. The experiments were scaled-up to 6-cm dishes using 1 × 10^6 ^cells and 1.5, 3.0 and 4.5 μg miRNA. For FABP5 silencing using siRNA, siGenome SMARTpool siRNA (Dharmacon) were used following the conditions described in the text.

### qRT-PCR analysis

Total RNA was isolated using Qiagen (Valencia, CA) RNeasy kit following the manufacturer's protocol. qRT-PCR was used to quantify mRNA levels of SREBP-1, FABP5 and β-actin using primer pairs: SREBP-1, 5'-TGCTTTGGAACCTCATCCGC-3' and 5'-AGCAGCAAGATGTCCTCCTGT-3'; FABP5, 5'-CCATGGCCAGCCTTAAGGA-3' and 5'-ACCTTCTCATAGACCCGAGT-3'; β-actin, 5'-CTACAATGAGCTGCGTGTGGC-3' and 5'-ATGGCTGGGGTGTTGAAGGTC-3'. qRT-PCR was performed using an ABI PRISM 7500 instrument (ABI, Foster City, CA) and SYBR Green Qiagen One-Step reverse transcription-PCR kit (Qiagen, Valencia, CA), following the manufacturer's recommendations. Quantification of mRNA levels was done by analyzing 50 ng of total RNA, in duplicate and comparing *C_t _*values with those of the standard curve. The β-actin gene was used as loading control. Fold changes are expressed relative to the shSCR-transfected group.

### Western blot

Transfected primary hepatocytes were lysed in modified RIPA buffer containing protease inhibitors, as previously described [22]. Protein concentration was determined using the BCA kit from Pierce (Rockford, IL). Proteins (20-40 μg) were separated in 4-20% SDS-polyacrylamide Criterion gels (Bio-Rad, Hercules, CA) and transferred to polyvinylidene difluoride (PVDF) membranes (Bio-Rad). Primary antibodies were used at the following concentrations in overnight incubations at 4°C: SREBP-1, 1:400; FABP5, 1:500; β-actin, 1:500). Secondary antibody was added and incubated at room temperature for 1 hour at the following dilutions: HRP-conjugated anti-rabbit IgG, 1:3,000; anti-goat IgG, 1:3,000. The anti-SREBP-1 and β-actin antibodies were purchased from Santa Cruz Biotechnology (Santa Cruz, CA). The anti-FABP5 and all secondary antibodies were purchased from R&D Systems (Minneapolis, MN). Blots were developed with Immun-Star (Bio-Rad) and exposed to ECL film (GE Healthcare).

### Statistical analysis

All experimental conditions were done in duplicate and repeated in at least two separate hepatocyte isolations. Data are presented as the arithmetic mean ± standard deviation. Statistical differences were calculated with a two-tailed, unpaired Student's t-test. Data were considered significant at p < 0.05.

## Abbreviations

siRNA: small interfering RNA; shRNA: short hairpin RNA; miRNA: microRNA; RNAi: RNA interference; FABP5: Fatty Acid Binding Protein 5; SREBP-1: Sterol Regulatory Element-Binding Protein-1.

## Competing interests

The authors declare that they have no competing interests.

## Authors' contributions

JSP - conducted transfection and molecular biology experiments with plasmids and siRNA, and wrote parts of the manuscript; SS - conducted transfection experiments with miRNA; LMK - assisted in setting up the primary hepatocyte isolation protocol; NM - designed, supervised and coordinated experiments, as well as wrote the paper. All authors have read and approved the manuscript.
